# Feasibility and effects of a multicomponent program incorporating esports on physical and cognitive function among older adults: A non-randomised pilot trial

**DOI:** 10.1371/journal.pone.0357666

**Published:** 2026-09-03

**Authors:** Yuta Nemoto, Fumie Otsuka, Shihoko Suzuki, Saori Kataoka, Tomoko Nakanishi, Takuya Ueda, Susumu Ogawa, Sumika Karasawa, Mikiko Shimaoka, Hiroto Narimatsu, Ung-il Chung

**Affiliations:** 1 School of Health Innovation, Kanagawa University of Human Services, Kawasaki, Kanagawa, Japan; 2 Tokyo Metropolitan Institute for Geriatrics and Gerontology, Itabashi, Tokyo, Japan; 3 School of Public Health, The University of Queensland, Brisbane, Queensland, Australia; 4 Center for Innovation Policy, Kanagawa University of Human Services, Kawasaki, Kanagawa, Japan; 5 School of Nutrition and Dietetics, Faculty of Health and Social Work, Kanagawa University of Human Services, Yokosuka, Kanagawa, Japan; 6 Center for Research Strategy, Waseda University, Shinjuku, Tokyo, Japan; 7 Cancer Prevention and Control Division, Kanagawa Cancer Center Research Institute, Yokohama, Kanagawa, Japan; İzmir Democracy University: Izmir Demokrasi Universitesi, TÜRKIYE

## Abstract

**Background:**

Esports (competitive and organised video gaming) may enhance physical and cognitive health in older adults. The aim of this study was to examine the feasibility and effects of a multicomponent program incorporating esports on physical and cognitive function in community-dwelling older adults.

**Methods:**

This parallel, non-randomised controlled trial was conducted in Yokosuka, Kanagawa, Japan. Twenty-six participants who 1) were aged 60 years or older, 2) had no cognitive impairment, 3) had no major health problems, and 4) had no prior experience with digital games were allocated to either the intervention (n = 12) or the control (n = 14) group. Participants in the intervention group attended weekly 90-minute sessions consisting of health education, exercise, and esports for 12 weeks. The feasibility of the program was assessed based on adverse events, participant retention, adherence to the program, engagement in digital games outside the program, enjoyment of the program, and willingness to continue playing esports after the study period. Additionally, psychological status, physical and cognitive function, and body composition were assessed.

**Results:**

No adverse events were reported; 10 of the 12 participants attended ≥80% of the sessions, all participants reported enjoying the program, and no participants dropped out during follow-up. Positive psychological status scores increased from before to after each session among participants in the intervention group. Furthermore, adjusted analyses showed more favourable changes in gait speed and balance in the intervention group than in the control group (*p* < 0.05). However, the effects on physical function remain uncertain because the analyses were exploratory.

**Conclusions:**

The program showed potential for high retention and adherence among older adults, with no adverse events. Larger-scale trials are needed to confirm the effectiveness of this intervention program, as the analyses in this study were exploratory.

**Trial registration:**

The study protocol was registered with the UMIN-CTR (ID: UMIN000055350; date of first registration: 27 August 2024).

## Introduction

The number of older adults living with dementia and functional disabilities has been increasing worldwide. This trend places not only a massive financial strain on society but also a significant physical burden on caregivers [1]. As the number of people affected is projected to grow rapidly [2], preventing these conditions has become a key public health priority.

Engagement in social group activities, such as sports and hobbies, promotes a socially and physically active lifestyle. Social participation helps prevent social isolation [3] and supports the maintenance or improvement of physical activity levels in older age [4], which, in turn, contributes to better physical and cognitive health and helps prevent functional disability and dementia [5,6]. Given its importance, social participation is included in the WHO Age-friendly Cities Framework [7]. In line with this, the Japanese government has promoted social group engagement among older adults, and the proportion of participants in such activities has been increasing [8].

However, the majority of these activities focus on exercise [8]. Previous studies have reported that participants in such activities tend to have better health and higher socioeconomic status compared to non-participants [9]. In contrast, older adults with poor health and lower socioeconomic status, such as those with less educational attainment, are less likely to participate. This may indicate that social group activities have not been equally accessible to these populations. Therefore, it is essential to develop alternative programs that appeal not only to older adults with better health and socioeconomic status but also to individuals who are unable or unwilling to attend exercise-based programs.

In recent years, the health effects of esports (competitive and organised video gaming) have attracted increasing attention [10,11]. Although some studies have reported that sedentary behaviours may be harmful to cognitive health [12,13], our previous research showed that mentally active sedentary behaviours are associated with a reduced risk of dementia onset [14]. Additionally, digital technology and exergames may improve physical and cognitive function [15,16]. Therefore, esports may have the potential to enhance physical and cognitive health in older adults [17]. Group-based esports may facilitate social interactions and enjoyment that are not necessarily available when digital games are played individually. Additionally, esports may have acute positive effects on psychological well-being, which could help foster positive relationships among participants and reduce dropout rates in multicomponent interventions that also include exercise and health education. Furthermore, game difficulty can be adapted to participants’ abilities, allowing individuals with varying levels of functional capability to enjoy playing together.

However, the effects of esports programs are not yet well understood, as most previous studies have applied observational or single-arm intervention designs [18,19]. The feasibility and effects of esports programs on health outcomes should be evaluated using a parallel-group trial design. Since providing multicomponent interventions to prevent dementia and functional disabilities is recommended [20–22], and esports may enhance the effects of exercise and health education, investigating the feasibility of a multicomponent program incorporating esports is important for developing effective health promotion strategies for community-dwelling older adults. The aim of this study was to examine the feasibility of a multicomponent program incorporating esports and to provide exploratory estimates of its effects on physical and cognitive function in community-dwelling older adults. The findings will inform the development of larger randomised controlled trials to determine the effects of the intervention on physical and cognitive health.

## Methods

### Study participants

This parallel, non-randomised controlled trial was conducted in Yokosuka, Kanagawa, Japan. From 30 August to 30 September 2024, we distributed flyers to residents living near the Yokosuka campus of Kanagawa University of Human Services and organised group sessions to explain the purpose and schedule of the study. The eligibility criteria were as follows: 1) aged 60 years or older, 2) without cognitive impairment (i.e., a score of at least 24 points on the Mini-Mental State Examination Japanese version [MMSE-J]) [23–25], 3) without major health problems, and 4) without prior experience with digital games. A total of 26 participants (12 in the intervention group and 14 in the control group) were recruited and completed the baseline assessment.

As there was no prior information available for a formal sample size calculation, we followed the recommendation of including 12 participants per group in a pilot study [26]. This number is based on considerations of feasibility, the precision of the mean and variance, and regulatory aspects [26]. Anticipating potential dropout in the control group during follow-up, we assigned 14 participants to the control group.

The protocol modification involved changes to the study design, allocation procedure, and sample size. The original protocol planned a larger randomised controlled trial based on an assumed moderate effect size. However, recruitment was delayed, and the number of participants available before the planned start date of the intervention was substantially lower than expected. Additionally, because of budget constraints, we were unable to extend the recruitment period or provide additional intervention sessions. We therefore revised the protocol to a non-randomised pilot trial in which group allocation was based on the registration period. Participants who registered during the first period (August 2024) were allocated to the intervention group, whereas those who registered during the second period (September 2024) were allocated to the control group. The resulting sample size was considered sufficient for assessing feasibility and estimating preliminary effects, but not to confirm efficacy.

As no formal consensus reporting guideline currently exists specifically for non-randomised pilot trials, we followed the Consolidated Standards of Reporting Trials (CONSORT) extension for randomised pilot and feasibility trials [27] (S1 Table). However, because this was a non-randomised pilot study, we did not report items that were not applicable to the study, in accordance with the recommendation of the guideline [28]. Instructions were provided both in person and via written materials, and all participants gave written informed consent at enrolment before the baseline assessment. This study was conducted in accordance with the Declaration of Helsinki. The study protocol was approved by the Ethics Committee of Kanagawa University of Human Services (Approval number: SHI-21) and registered in the UMIN-CTR (ID: UMIN000055350; date of first registration: 27 August 2024).

### Intervention program

In the intervention group, participants attended weekly 90-minute sessions held at the Yokosuka campus of Kanagawa University of Human Services from September 2024 to December 2024 (12 weeks). Each session consisted of three parts: 15 minutes of health education, 15 minutes of exercise, and 60 minutes of esports.

During the health education program, registered dietitians delivered lectures on nutrition and sleep, aiming to promote a healthy weight. The exercise program, delivered by occupational therapists, consisted of stretching and muscle strength training such as squats, calf raises, and standing side leg raises. Exercise intensity was adjusted based on each participant’s fitness level and perceived exertion.

In the esports program, participants played two games. The first game was Puyo Puyo (Sega Corp, Tokyo, Japan), a puzzle game in which players create groups of four or more Puyos of the same colour to make them disappear. Pairs of Puyos fall from the top of the screen in groups of two, and players can move and rotate them as they descend. Up to four people compete simultaneously, and a player loses when the Puyos reach a designated point at the top of the screen. To effectively erase Puyos, players must plan their placement and then move them according to their plan.

The second game was a rhythm-based game titled Taiko no Tatsujin (Bandai Namco Entertainment Inc., Tokyo, Japan). Players use a mini-Taiko drum designed for the game and hit the drum in time with the rhythm of a selected song. During the song, two types of commands, red and blue, appear from the right side of the screen. When the red command appears, players hit the face of the drum, and when the blue command appears, players hit the rim of the drum. Players earn points based on accuracy and timing, and the player with the highest score wins the game. To achieve a higher score, players need to identify each command and respond accurately.

Participants played Puyo Puyo during the first six weeks, followed by Taiko no Tatsujin for the remaining six weeks. During each session, four participants played while seated, and the others watched and cheered them on. The difficulty of these games, including aspects such as speed and complexity, was adjusted based on the participants’ performance levels.

Participants in the control group received the results of their baseline body composition assessments without any health guidance and then waited until the follow-up assessment was completed. After the follow-up assessment, they participated in the same intervention program as the intervention group, delivered over three weeks.

### Measurements

#### Feasibility assessment.

We assessed the feasibility of the program based on adverse events, participant retention, adherence to the program, engagement in digital games outside the program, enjoyment of the program, and willingness to continue playing esports after the study period. Four or five staff members per session observed each participant during the program to monitor for adverse events, and participants were asked to report any events, including illness, disability, or worsening of existing conditions, during the follow-up period. Participant retention was defined as completing both the program and the follow-up assessment.

For program adherence, we calculated the percentage of sessions attended and the proportion of participants who attended ≥80% of the sessions (i.e., 10 sessions). Higher adherence is associated with improved health outcomes [29], and 80% to 100% was considered high adherence in a previous review that examined the effects of exercise interventions on low back pain [30]. Thresholds for adherence (≥80%) and retention (≥80%) were predetermined based on a previous pilot study of an online physical activity intervention involving community-dwelling older adults [31]. Participants were also asked to report the reasons for any absences.

The frequency of playing digital games outside the program, enjoyment of the program, and willingness to continue playing esports were assessed using a questionnaire. Participants reported the frequency of playing digital games at home or in an arcade game centre, how much they enjoyed playing esports during the program, and how much they wanted to continue playing esports independently, without support from the research team. These self-report measures were developed for this study and have not been validated. Therefore, their reliability and validity remain unknown.

#### Psychological status.

Participants in the intervention group completed the Multiple Mood Scale Short Form [32] before and after each session. The scale consists of 8 domains, and we focused on 4 positive domains: liveliness, well-being, friendliness, and concentration. Each domain consists of 5 items, each rated on a 4-point Likert scale. We calculated the total score for each domain, ranging from 5 to 20. Higher scores indicated better psychological status.

#### Cognitive function.

Details of each measurement are presented in S2 Text. We assessed cognitive function, including global cognition, processing speed, visual memory, and planning, using the Japanese version of the Montreal Cognitive Assessment (MoCA-J) [33,34], the Trail Making Test (TMT) parts A and B [35], the Digit Symbol Substitution Test (DSST) [36], the Visual Memory Span Test (VMST) [37], and the Tower of Hanoi [38] (S2 Text). The assessors were blinded to group allocation.

#### Physical function.

Details of each measurement are shown in S3 Text. We assessed handgrip strength for muscle strength, the 5-meter walk test for gait speed [39], the single-leg stance test for balance ability, and the Timed Up and Go test (TUG) for functional mobility [40] (S3 Text). The assessors were not blinded to group allocation.

#### Body composition.

We assessed body weight, lean body mass, and body fat percentage using bioelectrical impedance analysis with the InBody 570 body composition analyser (InBody Co., Ltd, Seoul, South Korea), which has shown excellent agreement with dual-energy X-ray absorptiometry among middle-aged to older populations [41]. The assessors were not blinded to group allocation.

#### Sociodemographic factors.

We investigated sociodemographic factors such as age, sex, educational attainment (≤9 years, 10–12 years, or ≥13 years), subjective economic status (high, middle, or low), marital status (married or widowed/divorced/single), living arrangement (living alone or living with someone), employment status (full-time worker, part-time worker, or non-worker).

#### Subjective health conditions.

We included self-rated health (very good, good, fair, or poor), depressive symptoms, and higher-level functional capacity as subjective health conditions. The short form of the Geriatric Depression Scale [42], a 15-item measure, was used to assess depressive symptoms. The total score ranged from 0 to 15, with higher scores indicating more severe symptoms. For higher-level functional capacity, the TMIG index of competence, consisting of 13 items, was used [43]. The total score ranged from 0 to 13, with higher scores indicating a higher level of function.

### Statistical analysis

To examine differences in baseline characteristics between groups, we conducted Fisher’s exact tests for categorical variables and Welch’s t-tests for continuous variables.

For the assessment of changes in psychological conditions before and after each session, mixed-effects models with random intercepts were conducted using the R package “lme4” [44]. The models included week, time point (before or after the session), and their interaction terms as fixed effects. The coefficients for the time point indicated whether each psychological condition changed during the session, and the coefficients for the interaction terms indicated whether the changes in psychological status during the session increased or decreased over time.

Analysis of covariance (ANCOVA) was conducted to examine the effects of the program on health outcomes. Age, sex, and the baseline value of each outcome were adjusted for. Estimated marginal means were calculated based on ANCOVA using the R package “emmeans” [45] to present changes in each outcome during the follow-up. We also calculated partial eta-squared as the measure of effect size. Values of ≥0.01, ≥ 0.06, and ≥0.14 were interpreted as small, medium, and large effects, respectively [46]. The assumptions of ANCOVA were assessed as follows: the normality of residuals was evaluated using diagnostic plots and the Shapiro-Wilk test; the homogeneity of regression slopes assumption was assessed by testing the interaction between group and baseline outcome value; and the homoscedasticity was examined using Levene’s test. When the normality of the residuals was not supported, the baseline and follow-up outcome values were log-transformed (i.e., TMT-B, DSST, and Tower of Hanoi). When the assumption of homogeneity of regression slopes was not supported, a linear regression model including group, the mean-centred baseline outcome value, and their interaction was fitted (i.e., for the TUG). Partial R-squared values were calculated for each model term as measures of effect size after adjustment for the other terms included in the model. Values of 0.02, 0.13, and 0.26 were used as reference points for small, medium, and large effects, respectively [47].

In terms of missing values, one participant refused to complete the Tower of Hanoi at the baseline assessment, and we excluded data from this participant from the analysis examining the change in the Tower of Hanoi. There were no missing values in the other variables.

All analyses were conducted using R 4.5.1 (R Foundation for Statistical Computing, Vienna, Austria).

## Results

**Fig 1:**
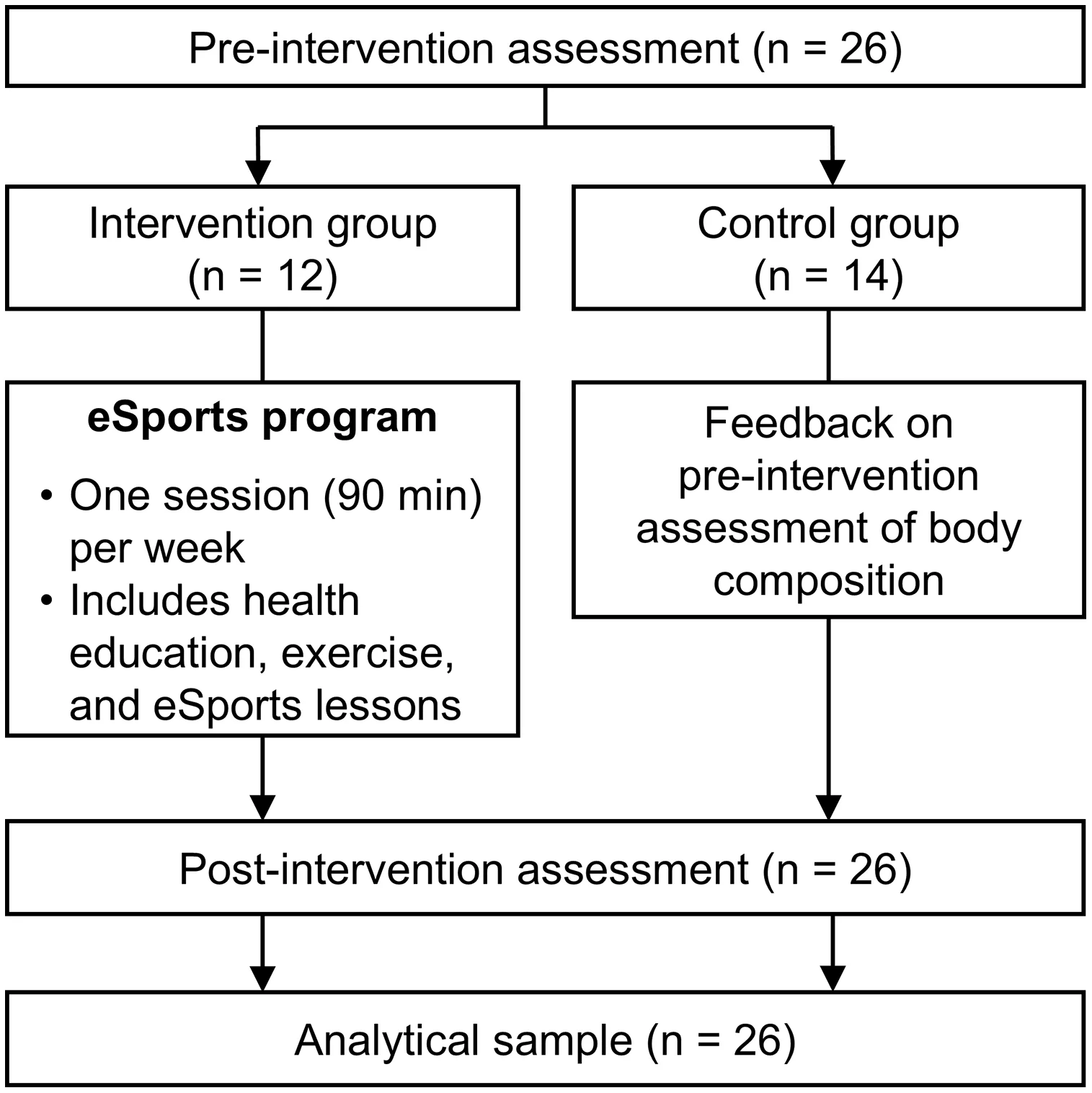
Study design and participant flow.

The flow of this study is shown in Fig 1. After the baseline assessment, 26 older adults who met the eligibility criteria were allocated to the intervention (n = 12) or the control group (n = 14). All participants completed the intervention and the follow-up assessment and were included in the analyses (Fig 1).

The baseline characteristics of participants in the intervention and control groups are shown in Table 1. There were significant differences in the MoCA-J, TMT-A, gait speed, and single-leg stance test. Participants in the control group demonstrated better cognitive and physical function at baseline (Table 1).

**Table 1:** Baseline characteristics of participants in each group.

	Intervention group (n = 12)	Control group (n = 14)	*p-value*
**Age** – mean (SD)	70.2 (5.1)	71.6 (4.3)	0.46
**Sex** – n (%)			
Male	1 (8.3)	3 (21.4)	0.60
Female	11 (91.7)	11 (78.6)	
**Educational attainment** – n (%)			
≤ 9 years	2 (16.7)	0 (0.0)	0.25
10–12 years	6 (50.0)	6 (42.9)	
≥ 13 years	4 (33.3)	8 (57.1)	
**Subjective economic status** – n (%)			
High	7 (58.3)	9 (64.3)	1.00
Middle	5 (41.7)	5 (35.7)	
Low	0 (0.0)	0 (0.0)	
**Marital status** – n (%)			
Married	8 (66.7)	8 (57.1)	0.70
Widowed/Divorced/Single	4 (33.3)	6 (42.9)	
**Living arrangement** – n (%)			
Living alone	3 (25.0)	3 (21.4)	1.00
Living with someone	9 (75.0)	11 (78.6)	
**Employment status** – n (%)			
Full-time worker	1 (8.3)	2 (14.3)	1.00
Part-time worker	5 (41.7)	5 (35.7)	
Non-worker	6 (50.0)	7 (50.0)	
**Self-rated health** – n (%)			
Very good	4 (33.3)	1 (7.1)	0.15
Good	8 (66.7)	13 (92.9)	
Fair/Poor	0 (0.0)	0 (0.0)	
**Depressive symptoms** – mean (SD)	2.8 (3.4)	1.9 (1.7)	0.42
**Higher-level functional capacity** – mean (SD)	11.3 (1.4)	11.6 (0.8)	0.49
**Cognitive function**			
**MoCA-J** – mean (SD)	23.8 (2.5)	25.9 (1.7)	0.02
**TMT-A** – mean (SD)	46.3 (10.2)	36.5 (8.2)	0.01
**TMT-B** – mean (SD)	102.0 (35.8)	92.1 (37.0)	0.49
**VMST (forward)** – mean (SD)	8.3 (1.5)	9.1 (1.5)	0.15
**VMST (backward)** – mean (SD)	8.4 (1.4)	8.3 (1.4)	0.82
**VMST (total)** – mean (SD)	16.7 (2.3)	17.4 (2.5)	0.43
**DSST** – mean (SD)	66.3 (10.9)	75.7 (17.0)	0.10
**Tower of Hanoi** – mean (SD)	9.6 (4.0)	8.6 (2.4)	0.45
Missing	1	0	
**Physical function**			
**Grip strength (kg)** – mean (SD)	25.7 (4.4)	26.7 (6.6)	0.63
**Five-meter walk test (m/s)** – mean (SD)	1.8 (0.3)	2.2 (0.3)	0.01
**Single-leg stance test (s)** – mean (SD)	21.4 (17.8)	39.7 (22.7)	0.03
**Timed Up and Go (s)** – mean (SD)	6.0 (1.4)	5.6 (1.0)	0.42
**Body composition**			
**Body weight** – mean (SD)	59.1 (12.0)	55.8 (10.2)	0.46
**Lean body mass** – mean (SD)	36.4 (4.9)	37.6 (8.3)	0.65
**Body fat percentage** – mean (SD)	33.5 (7.2)	28.2 (6.6)	0.07

MoCA-J = Montreal Cognitive Assessment Japanese version, TMT = Trail Making Test, VMST = Visual Memory Span Test, DSST = Digit Symbol Substitution Test

Group differences were examined using Fisher’s exact tests for categorical variables and Welch’s t-test for continuous variables.

The feasibility of the multicomponent program incorporating esports is shown in Table 2. Among participants in the intervention group, no adverse events were reported, and no one dropped out during the follow-up period. Of the 12 participants, 10 (83.3%) attended ≥80% of the sessions. The primary reasons for absence were work or household responsibilities, such as caring for family members. Regarding engagement in digital games outside the program, 11 participants (91.7%) did not play any games. All participants reported enjoying the intervention program, but only six (50%) expressed a willingness to continue playing esports independently (Table 2).

**Table 2:** Feasibility of the multicomponent program incorporating esports.

	Intervention group (n = 12) n (%)
**Adverse events**	0 (0.0)
**Retention**	12 (100.0)
**Adherence (attended sessions [%])**	
8 [67%]	1 (8.3)
9 [75%]	1 (8.3)
10 [83.3%]	4 (33.3)
11 [91.7%]	4 (33.3)
12 [100%]	2 (16.7)
**Engagement in digital games outside the program**	
More than once a month	0 (0.0)
Once a month	1 (8.3)
None	11 (91.7)
**Enjoyment of the program**	
Very enjoyable	8 (66.7)
Relatively enjoyable	4 (33.3)
Other	0 (0.0)
**Willingness to continue playing esports**	
Agree	6 (50.0)
Neither agree nor disagree	5 (41.7)
Disagree	1 (8.3)

**Fig 2:**
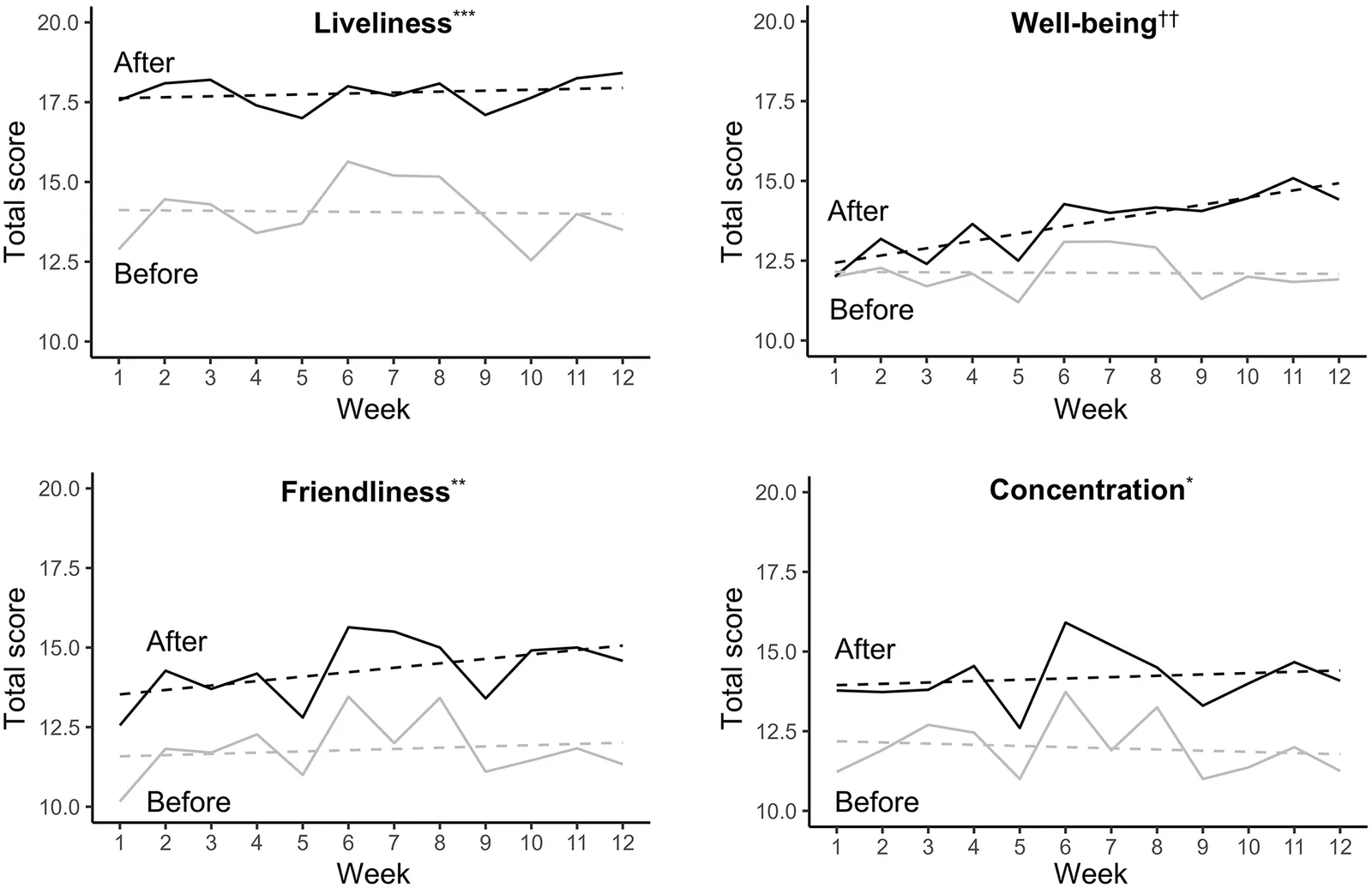
Positive psychological status before and after each session among participants in the intervention group (n = 12). Mixed-effects models with random intercepts were conducted. The models included week as a timescale variable, time point (i.e., before or after the session), and their interaction terms with the timescale as the independent variables. * *p* < 0.05 for time point, ** *p* < 0.01 for time point, *** *p* < 0.001 for time point. ^†^*p* < 0.05 for interaction term, ^††^*p* < 0.01 for interaction term, ^†††^*p* < 0.001 for interaction term.

Changes in psychological status before and after each session among participants in the intervention group are presented in Fig 2. For liveliness, friendliness, and concentration, scores after the sessions were higher than those before the sessions (liveliness: *p* < 0.001, friendliness: *p* = 0.004, concentration: *p* = 0.01). For well-being, the differences between pre- and post-session scores widened over time (interaction between timescale and time point: *p* = 0.005) (Fig 2).

**Fig 3:**
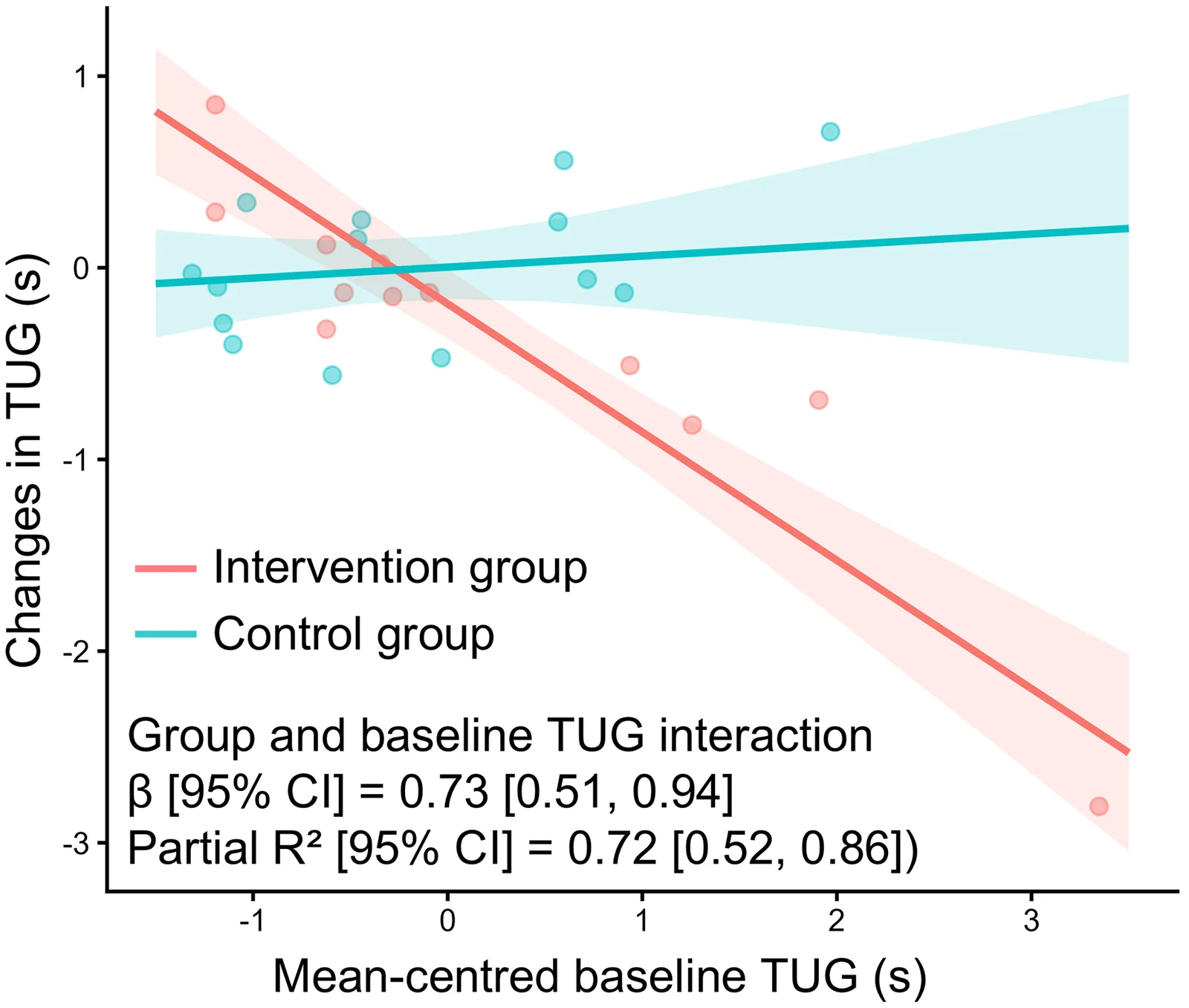
Comparison of changes in Timed Up and Go test performance between the intervention and control groups (n = 26).

Comparisons of changes in outcomes between the intervention and control groups are shown in Table 3 and Fig 3. Compared with participants in the control group, those in the intervention group showed more favourable changes, with large effect sizes (≥0.14 partial eta-squared), in gait speed (5-meter walk test: *p* < 0.001) and balance ability (Single-leg stance test: *p* = 0.01). However, the CIs for the effect size estimates were very wide. Although group differences in body weight and body fat percentage did not reach statistical significance, both outcomes showed large effect sizes (Table 3). For the TUG, the assumption of homogeneity of regression slopes was not met. Therefore, a linear regression model including an interaction term between group and the baseline outcome value was fitted. Although the main effect of group at the mean baseline TUG was not significant (β [95% CI] = 0.19 [−0.06, 0.45]), a significant interaction between group and baseline TUG was observed (β [95% CI] = 0.73 [0.51, 0.94], partial R-squared [95% CI] = 0.72 [0.52, 0.86]), indicating that the association between baseline TUG and changes in TUG differed between the groups (Fig 3).

**Table 3:** Comparison of changes in outcomes between the intervention and control groups (n = 26).

	Intervention group (n = 12) Change [95% CI]	Control group (n = 14) Change [95% CI]	*p-value*	Effect size Partial η² [95% CI]
**Cognitive function**				
MoCA-J	2.1 [0.4, 3.7]	2.1 [0.6, 3.5]	0.09	0.13 [0.00, 1.00]
TMT-A	−6.0 [−14.6, 2.6]	−4.1 [−11.5, 3.4]	0.20	0.08 [0.00, 1.00]
TMT-B*	4.5 [−19.5, 28.4]	−11.7 [−31.4, 8.0]	0.14	0.10 [0.00, 1.00]
VMST (forward)	−0.1 [−1.2, 1.0]	0.0 [−1.0, 0.9]	0.41	0.03 [0.00, 1.00]
VMST (backward)	−0.1 [−1.1, 0.8]	−0.1 [−0.9, 0.7]	0.90	<0.01 [0.00, 1.00]
VMST (total)	−0.3 [−1.9, 1.4]	−0.1 [−1.6, 1.4]	0.55	0.02 [0.00, 1.00]
DSST*	1.0 [−3.7, 5.7]	4.4 [0.6, 8.3]	0.20	0.08 [0.00, 1.00]
Tower of Hanoi*	−3.2 [−12.5, 6.1]	3.2 [−4.8, 11.2]	0.28	0.06 [0.00, 1.00]
**Physical function**				
Grip strength (kg)	0.76 [−0.52, 2.03]	1.04 [−0.08, 2.16]	0.42	0.03 [0.00, 1.00]
Five-meter walk test (m/s)	0.01 [−0.10, 0.12]	−0.08 [−0.18, 0.01]	<0.001	0.55 [0.29, 1.00]
Single-leg stance test (s)	0.4 [−9.9, 10.8]	−5.4 [−14.6, 3.7]	0.01	0.27 [0.04, 1.00]
**Body composition**				
Body weight (kg)	0.24 [−0.77, 1.26]	1.04 [0.24, 1.83]	0.05	0.17 [0.00, 1.00]
Lean body mass (kg)	0.02 [−0.84, 0.89]	−0.14 [−0.82, 0.54]	0.56	0.02 [0.00, 1.00]
Body fat percentage (%)	0.16 [−1.50, 1.82]	1.45 [0.15, 2.75]	0.06	0.16 [0.00, 1.00]

Change = adjusted mean change from baseline, CI = confidence interval, MoCA-J = Montreal Cognitive Assessment Japanese version, TMT = Trail Making Test, VMST = Visual Memory Span Test, DSST = Digit Symbol Substitution Test

Analysis of covariance (ANCOVA) was conducted. Age, sex, and the baseline value of each outcome were adjusted. Estimated marginal means were calculated based on ANCOVA to present changes in each outcome during the follow-up. All *p-values* are exploratory and were not adjusted for multiple comparisons.

*Log transformation was applied because the assumption of residual normality was not met.

Effect size: Partial eta-squared. It ranges from 0 to 1, and values ≥0.01 indicate a small effect, ≥ 0.06 indicate a medium effect, and ≥0.14 indicate a large effect.

The Tower of Hanoi analysis included 25 participants (intervention group, n = 11; control group, n = 14).

A linear regression model including group, mean-centred baseline TUG, and their interaction term was fitted because the assumption of homogeneity of regression slopes was not met. Age and sex were included as covariates. Change was calculated as the follow-up value minus the baseline value; therefore, negative values indicate improvement. Points represent observed values, lines represent model-predicted values, and shaded areas represent 95% confidence intervals.

## Discussion

This study examined the feasibility of a multicomponent program incorporating esports and provided exploratory estimates of its effects on physical and cognitive function among community-dwelling older adults. The main findings were that the program was feasible, with no adverse events, no loss to follow-up, high adherence, and high reported enjoyment. Exploratory analyses also suggested more favourable changes in physical function, including walking speed, single-leg standing time, and the TUG performance. In contrast, no clear improvement in cognitive function was observed. Given the small sample size and non-randomised design, these preliminary findings should be interpreted with caution and examined in a larger randomised controlled trial.

Our findings suggest that participants enjoyed esports during the program. The games selected for this study were easy to play, even for older adults, and their difficulty was adjusted based on the participants’ performance levels. As a result, participants could perceive improvements in their skills, were not overwhelmed by the games, and enjoyed them from the first session. However, the participants rarely played outside the program, and only half expressed a willingness to continue playing esports independently. Although the reasons for this were not investigated, one possible explanation is the need to purchase and set up a video game console, gaming monitor, and software. This possibility should be examined in future studies. Additionally, participants did not appear to become accustomed to playing esports independently, as few played at home, and only half were willing to continue playing esports after the intervention period. However, given that most participants enjoyed the program and that retention and adherence were high, its dissemination and implementation may be feasible with ongoing but less intensive support from researchers, local governments, and volunteers.

Within the intervention group, scores for all domains of positive psychological status increased from before to after the sessions, and the pre-post difference in well-being increased over time. These findings are consistent with those of a previous intervention study that examined the acute effects of esports on emotional states [19]. While the previous study conducted only a single session, this study assessed within-session changes over 12 sessions and observed sustained increments. This may reflect the development of social relationships among participants during the intervention period and the enjoyment they derived from attending the weekly sessions. These results suggest that the program may enhance positive psychological status in older adults throughout the intervention period. However, because these outcomes were not assessed in the control group, expectancy and novelty effects cannot be ruled out.

In terms of cognitive function, the effect size for global cognition was slightly below the threshold for a large effect. However, the CI was very wide, and the estimate was unstable because of the small sample size. In addition, the estimated marginal means for the MoCA-J, adjusted for age, sex, and baseline outcome values, were identical between the groups. These findings indicate that the effect of the program on global cognition remains uncertain. Although processing speed and planning showed medium effect sizes, the group differences were not statistically significant. One possible explanation is that the participants were relatively young and had relatively high cognitive function at baseline, which may have limited the potential for improvement. In addition, the intervention period may have been too short to produce detectable improvements in cognitive function. Large-scale intervention trials with longer follow-up periods are needed to determine the effects of the program on cognitive function.

For physical function, large effect sizes were observed in gait speed, balance, and functional mobility. Many previous studies have reported that resistance exercise is beneficial for improving physical function, including gait speed and functional mobility [48]. In the intervention, the exercise component focused on improving lower-body function. Additionally, participants in the intervention group had opportunities to go out weekly, which may have increased their physical activity levels and maintained physical function, except for handgrip strength. However, since these potential mechanisms are speculative and the effects of individual components of a multicomponent intervention are difficult to disentangle, further studies are needed. The significant interaction term observed in the TUG analysis should be interpreted with caution because of the small sample size. The intervention group included one participant with poor TUG performance at baseline whose performance improved substantially at the follow-up assessment. This observation may have influenced the results. Therefore, the finding should be examined in a larger randomised controlled trial.

Regarding body composition, large effect sizes were observed for body weight and body fat percentage, but they did not reach statistical significance. Participants in the control group may have gained more weight and body fat than those in the intervention group. A previous longitudinal study among Japanese older adults reported seasonal increases in body weight and body fat percentage during winter compared to summer [49]. In the multicomponent program incorporating esports, which was conducted from September to December, registered dietitians provided health education aimed at maintaining a healthy weight. This may have contributed to a reduction in seasonal weight gain in the intervention group. However, given the very wide CIs, unstable estimates, and non-significant group differences, the effects of the intervention on body composition remain uncertain.

### Strengths and limitations

The strength of this study lies in the comprehensive range of measurements used to assess cognitive function. This allowed us to examine which aspects of cognitive function might be improved by the intervention program. Another strength is the assessment of psychological status before and after each session, enabling us to examine both within-session changes across the 12-week intervention period among participants in the intervention group.

Several limitations must be considered when interpreting the results. First, this study was a non-randomised controlled trial, and there were several differences in baseline characteristics between the intervention and control groups. Although we adjusted for baseline values in the statistical models, the effects of the intervention could not be conclusively determined because this adjustment may not have fully accounted for differences in baseline characteristics. Additionally, regression to the mean may have contributed to the differences between groups. For example, some participants may have shown lower-than-actual physical function at baseline due to temporary poor health and then returned to their usual level of function at follow-up. Furthermore, while participants who registered earlier were unaware that they would be allocated to the intervention group, those who registered later knew that they would be allocated to the control group. This allocation procedure may have increased the risk of selection bias. Second, this was a pilot study with a small sample size, and its primary purpose was to examine the feasibility of the program. Therefore, the validity and reproducibility of the findings regarding its health effects are limited. Additionally, because the assessors of physical function and body composition were not blinded, assessor bias may have affected these outcomes. Larger-scale intervention studies are required to confirm these findings. Third, since the participants were relatively young and healthy older adults, and most were women, the generalisability of our findings to other populations, such as older men with lower levels of functional capability, may be limited. Fourth, because several cognitive measures were conducted repeatedly over a relatively short period, practice effects may partly explain the improvements in cognitive function observed in both groups. However, because the same assessment schedule was used for both groups, practice effects are unlikely to fully explain the observed group differences. Fifth, as this was an exploratory pilot study, adjustment for multiple comparisons was not performed. Therefore, the findings should be interpreted with caution due to an increased risk of a Type I error. Finally, the feasibility measures have not been validated, and their reliability and validity remain unknown.

## Conclusions

It is essential to develop intervention programs that enhance the health of older adults while ensuring a high level of safety. Although it is challenging to isolate the effects of individual components of a multicomponent intervention, such interventions have provided stronger evidence for preventing cognitive decline than isolated interventions [22,50]. The multicomponent program incorporating esports achieved high retention and adherence among older adults, with no adverse events reported. Although exploratory analyses suggested possible changes in physical function outcomes, the effects of the intervention remain uncertain and should be examined in a larger, high-quality randomised controlled trial.

## Supporting information

S1 Table CONSORT 2010 checklist of information to include when reporting a pilot or feasibility trial. (DOCX)

S2 Text Measurements of cognitive function. (DOCX)

S3 Text Measurements of physical function. (DOCX)
