# Supplementary material for: Feasibility and effects of a multicomponent program incorporating esports on physical and cognitive function among older adults: A non-randomised pilot trial
Source: PLoS One. 2026 Sep 3;21(9):e0357666. doi: 10.1371/journal.pone.0357666 (PMC13541156; doi:10.1371/journal.pone.0357666)
Supplement: Supplementary file 2 [file pone.0357666.s002.docx]

**S2 Text. Measurements of cognitive function**

We conducted the Mini-Mental State Examination (MMSE) (1, 2) to select participants without cognitive impairment at baseline. Scores range from 0 to 30, with higher scores indicating better cognitive function. We used a cutoff of 24 points based on a previous study (3).

To examine the effects of the intervention program on cognitive function, including global cognition, processing speed, visual memory, and planning, we used the Japanese version of the Montreal Cognitive Assessment (MoCA-J) (4, 5), the Trail Making Test (TMT) parts A and B (6), the Digit Symbol Substitution Test (DSST) (7), the Visual Memory Span Test (VMST) (8), and the Tower of Hanoi (9).

The MoCA-J measures global cognition, including attention, concentration, executive function, memory, language, visuoconstructional skills, conceptual thinking, calculations, and orientation. The score ranges from 0 to 30, with higher scores indicating better cognitive function. The cutoff for mild cognitive impairment (MCI) is 26 points (5). This measure has been reported to have high sensitivity and specificity for detecting MCI among community-dwelling older adults (5).

For the assessment of processing speed, we used TMT-A, TMT-B, and the DSST. In TMT-A, participants were asked to connect numbers from 1 to 25, which were randomly arranged, in ascending order. In TMT-B, participants connected numbers (from 1 to 13) and Japanese Hiragana letters (12 letters) alternately. Participants completed both tests as quickly as possible, and the time taken was recorded (6).

In DSST, nine numbers (from 1 to 9) and corresponding symbols (each assigned to a specific number) were provided to participants. They were asked to fill in the blanks with symbols corresponding to the numbers within 2 minutes. The number of correct answers was used as the score (7).

We assessed visual memory using the VMST (8) from the Wechsler Memory Scale-Revised. This measure consists of forward and backward assessments. In the forward assessment, the assessors touched squares printed on paper in a random order, and participants were asked to touch the squares in the same order. In the backward assessment, participants were asked to touch the squares in the reverse order of the assessor. The score for each assessment ranges from 0 to 14, and the total score ranges from 0 to 28.

To assess the planning ability, we used the Tower of Hanoi (9). It is a puzzle consisting of three pegs and several disks of varying size. Given a starting state, in which the disks were stacked on one or more pegs, the task was to reach a goal state in which the disks were stacked in descending order on a specified peg. There were three constraints on moving the disks. (1) Only one disk can be moved at a time. (2) Any disk not being moved must remain on a peg. (3) A larger disk cannot be placed on a smaller disk. We used No. 6 from the previous study (9), and recorded the number of moves as the score.

**Reference**

1.	Folstein MF, Folstein SE, McHugh PR. "Mini-mental state". A practical method for grading the cognitive state of patients for the clinician. J Psychiatr Res. 1975;12(3):189–98.

2.	Sugishita M, Koshizuka Y, Sudou S, Sugishita K, Hemmi I, Karasawa H, et al. The Validity and Reliability of the Japanese Version of the Mini-Mental State Examination （MMSE-J） with the original procedure of the Attention and Calculation Task（2001）. Japanese Journal of Cognitive Neuroscience. 2018;20(2):91–110.

3.	Sakurai R, Kim Y, Inagaki H, Tokumaru AM, Sakurai K, Shimoji K, et al. MMSE Cutoff Discriminates Hippocampal Atrophy: Neural Evidence for the Cutoff of 24 Points. Journal of the American Geriatrics Society. 2021;69(3):839–41.

4.	Nasreddine ZS, Phillips NA, Bédirian V, Charbonneau S, Whitehead V, Collin I, et al. The Montreal Cognitive Assessment, MoCA: a brief screening tool for mild cognitive impairment. J Am Geriatr Soc. 2005;53(4):695–9.

5.	Fujiwara Y, Suzuki H, Yasunaga M, Sugiyama M, Ijuin M, Sakuma N, et al. Brief screening tool for mild cognitive impairment in older Japanese: Validation of the Japanese version of the Montreal Cognitive Assessment. Geriatrics and Gerontology International. 2010;10:225–32.

6.	Llinàs-Reglà J, Vilalta-Franch J, López-Pousa S, Calvó-Perxas L, Torrents Rodas D, Garre-Olmo J. The Trail Making Test:Association With Other Neuropsychological Measures and Normative Values for Adults Aged 55 Years and Older From a Spanish-Speaking Population-Based Sample. Assessment. 2017;24(2):183–96.

7.	D. Wechsler. Wechsler Adult Intelligence Scale - Fourth Edition 2008 [cited 2025 20 July]. Available from: <https://www.pearsonassessments.com/en-us/Store/Professional-Assessments/Cognition-%26-Neuro/Wechsler-Adult-Intelligence-Scale-%7C-Fourth-Edition/p/100000392?srsltid=AfmBOoq8Adkc1rrzNYvkqigR3lbxIH4MVaHuuvXRRwaaesGx8ajC4lXN>.

8.	Wechsler D. WMS-R: Wechsler Memory Scale--Revised : Manual: Psychological Corporation; 1987.

9.	Goel V, Grafman J. Are the frontal lobes implicated in "planning" functions? Interpreting data from the Tower of Hanoi. Neuropsychologia. 1995;33(5):623–42.
