# Supplementary material for: Feasibility and effects of a multicomponent program incorporating esports on physical and cognitive function among older adults: A non-randomised pilot trial
Source: PLoS One. 2026 Sep 3;21(9):e0357666. doi: 10.1371/journal.pone.0357666 (PMC13541156; doi:10.1371/journal.pone.0357666)
Supplement: Supplementary file 3 [file pone.0357666.s003.docx]

**S3 Text. Measurements of physical function**

Muscle strength was measured by assessing handgrip strength using a Smedley dynamometer (T-1781, Toei Light Corp, Saitama, Japan). Handgrip strength was evaluated twice on each hand. Participants were asked to inhale deeply and exhale fully while squeezing the device with as much force as possible. The maximum value was used for analysis.

Gait speed was assessed using the 5-meter walk test. Participants were asked to walk along an 11-meter walkway as fast as possible twice. Velocity was calculated using the middle 5 meters of the 11-meter walkway. The ﬁrst and last 3 meters were considered acceleration and deceleration phases and were not included in the calculation. The fastest time was then used to calculate gait speed (m/s) (1).

Balance ability was evaluated using the single-leg stance test. Participants were asked to stand on one leg, keeping their eyes open and focusing on a mark at eye level. The assessors measured the time from when the participant’s leg left the floor until the foot touched down or the time reached 60 seconds. If the first attempt did not reach 60 seconds, the second attempt was allowed. The better record was used for the analysis.

To assess functional mobility, we used the Timed Up and Go test. Participants, starting from a seated position, were asked to stand up, walk 3 meters, turn around, walk back to the chair, and sit down as fast as possible. Assessors measured the time taken to complete the procedure (2). Participants completed this twice, and the fastest time was used.

**References**

1.	Sakurai R, Fujiwara Y, Yasunaga M, Takeuchi R, Murayama Y, Ohba H, et al. Regional Cerebral Glucose Metabolism and Gait Speed in Healthy Community-Dwelling Older Women. The Journals of Gerontology: Series A. 2014;69(12):1519–27.

2.	Podsiadlo D, Richardson S. The timed "Up & Go": a test of basic functional mobility for frail elderly persons. J Am Geriatr Soc. 1991;39(2):142–8.
